# Mitochondrial Abundance and Function Differ Across Muscle Within Species

**DOI:** 10.3390/metabo14100553

**Published:** 2024-10-16

**Authors:** Con-Ning Yen, Jocelyn S. Bodmer, Jordan C. Wicks, Morgan D. Zumbaugh, Michael E. Persia, Tim H. Shi, David E. Gerrard

**Affiliations:** School of Animal Sciences, Virginia Polytechnic Institute and State University, Blacksburg, VA 24061, USA; yencn17@vt.edu (C.-N.Y.); jocelynb@vt.edu (J.S.B.); jwicks4@unl.edu (J.C.W.); mdzumbaugh@ksu.edu (M.D.Z.); mpersia@vt.edu (M.E.P.); haoshi@vt.edu (T.H.S.)

**Keywords:** mitochondria, skeletal muscle, metabolism

## Abstract

*Background*: Mitochondria are considered the powerhouse of cells, and skeletal muscle cells are no exception. However, information regarding muscle mitochondria from different species is limited. *Methods*: Different muscles from cattle, pigs and chickens were analyzed for mitochondrial DNA (mtDNA), protein and oxygen consumption. *Results*: Bovine oxidative muscle mitochondria contain greater mtDNA (*p* < 0.05), protein (succinate dehydrogenase, SDHA, *p* < 0.01; citrate synthase, CS, *p* < 0.01; complex I, CI, *p* < 0.05), and oxygen consumption (*p* < 0.01) than their glycolytic counterpart. Likewise, porcine oxidative muscle contains greater mtDNA (*p* < 0.01), mitochondrial proteins (SDHA, *p* < 0.05; CS, *p* < 0.001; CI, *p* < 0.01) and oxidative phosphorylation capacity (OXPHOS, *p* < 0.05) in comparison to glycolytic muscle. However, avian oxidative skeletal muscle showed no differences in absolute mtDNA, SDHA, CI, complex II, lactate dehydrogenase, or glyceraldehyde 3 phosphate dehydrogenase compared to their glycolytic counterpart. Even so, avian mitochondria isolated from oxidative muscles had greater OXPHOS capacity (*p* < 0.05) than glycolytic muscle. *Conclusions*: These data show avian mitochondria function is independent of absolute mtDNA content and protein abundance, and argue that multiple levels of inquiry are warranted to determine the wholistic role of mitochondria in skeletal muscle.

## 1. Introduction

A major goal of animal agriculture, especially meat production, is to maximize lean protein accretion to increase food resources for the burgeoning global population at optimal efficiency and sustainability. Armed with this long-term goal, animal growth rate and feed efficiency has increased dramatically in all livestock species over the past 50 years [1,2]. Much of this improvement is a direct result of advances in animal management strategies, development of highly-robust genetic selection programs, and fortified diets, which have culminated in a reduced time to market weight [3]. In fact, time to harvest has decreased in broiler production to just over 30 days, while time to reach market weight for pigs and cattle can be as short as 5 and 15 months, respectively; meanwhile, market weights continue to increase [3,4,5]. While biological differences across species exist, especially regarding size—which intuitively explains some improvements in growth rate—differences in muscle metabolism also exist across these primary meat-producing species [6]. Moreover, purely from a feed efficiency standpoint, carbon sequestration reigns as the primary endpoint for those interested in improving animal growth efficiency, and likely contributes to differences in feed efficiencies across these animal species. This raises the question of whether observed changes in growth rate and feed efficiencies is related to changes in metabolism. Finally, mitochondria are pivotal to cellular metabolism. Substrates entering the mitochondria can be completely oxidized to create ATP for various cellular functions. However, full substrate oxidation results in the production of CO_2_, with an overall detriment to carbon sequestration and a potential loss to overall feed efficiency, which is a highly coveted key production indicator for the animal industries. Therefore, understanding the role of the muscle mitochondria across vastly different species and muscle types may provide insight into mechanisms controlling overall production efficiencies and sustainable food production.

Skeletal muscle is mostly a collection of muscle cells differing in their ability to contract and provide energy to support this unique function. As a result, whole muscle function is predicated on the relative contribution of various muscle fiber types and their associated metabolic profiles within the tissue [7]. Glycolytic muscles, also referred to as fast-twitch or white muscles, are often composed predominately of muscle fibers possessing type II myosin heavy chain (MyHC). In contrast, slow-twitch or red muscles are oxidative and composed primarily of type I MyHC, although type IIa fibers are generally considered oxidative fibers [8]. As the name implies, glycolytic muscles preferentially use glycolytic metabolism to generate ATP, whereas oxidative muscles rely on oxidative phosphorylation as the major energy source for ATP production [9,10]. Although glycolytic muscles rely mostly on glycolysis for energy production, they still contain mitochondria [11]. Consistent with this line of thought, Glancy and Balaban [12] showed that the protein composition of mitochondria from red and white muscles are similar, suggesting that mitochondria number is the primary determinant responsible for metabolic differences between glycolytic and oxidative muscles. However, these studies failed to assess other functional disparities in the mitochondria across distinct muscle types.

Across livestock species, faster growing, more efficient animals tend to have more glycolytic muscle, while slower growth rate in cattle correlates with a shift to more oxidative metabolism, as defined by greater oxidative enzyme abundance in muscles [13,14,15]. In pigs, domestication and selection for increased meat-producing ability and growth rate have changed muscle composition to a more glycolytic phenotype, at least in comparison to their wild pig predecessor [16]. Additionally, chickens primarily used for meat production have greatly reduced mitochondria in the *pectoralis* (breast) muscle that relies mostly on glycolytic metabolism. Likewise, chicken muscle mitochondria are influenced by breed and domestication; specifically, high-performing, fast-growing phenotypes contain less mitochondrial protein compared to their wildtype counterparts [17]. While the aforementioned association between lean growth rate and feed efficiency, and fiber type composition lacks a clear cause and effect relationship, more definitive data linking muscle type to lean growth efficiency were recently reported by Zeng et al. [18] using MyHC isoform knockout mice. These researchers showed mice lacking the ability to express type IIb MyHC, and thereby the fastest-contracting muscle fiber phenotype, resulted in mice incapable of responding to beta-adrenergic agonist stimulation and the presence of the myostatin null-induced muscle hypertrophy; two well-known stimulators of efficient, fast-contracting, glycolytic muscle. These data suggest type II glycolytic fibers are requisite for muscle hypertrophy and improved lean accretion and animal growth rate. Together, these data postulate that differences in mitochondrial function regardless of species warrant greater investigation. Therefore, the objective of the current study was to assess mitochondria differences across muscles within three agriculturally relevant species, to address differences in mitochondrial characteristics across species and muscle type, and possibly linking these differences to changes in animal production efficiency.

## 2. Materials and Methods

### 2.1. Animals and Sampling

Commercially raised cattle (*n* = 6), pigs (*n* = 6), and chickens (*n* = 10) were harvested on separate days at market weight (590 kg, 120 kg, and 3 kg, respectively) at the Virginia Tech Meat Center and Virginia Tech Poultry Farm (Blacksburg, VA, USA), respectively, following the standard procedures and in accordance with Virginia State Inspection. Muscle samples from a glycolytic and oxidative phenotype were excised approximately 5 min post exsanguination for each species. The *longissimus lumborum* (LL) and *masseter* (MS) were collected for both bovine and porcine species, while the *pectoralis major* (PM) and *quadriceps femoris* (QF) were collected from chickens. Regardless of species, or muscles collected, all samples were immediately snap frozen in liquid nitrogen and stored at −80 °C. Additionally, fresh tissue was also collected at this time, and immediately processed for mitochondrial isolation.

### 2.2. Mitochondrial DNA Copy Number

Mitochondrial DNA (mtDNA) was quantified using real-time polymerase chain reactions (qPCR), as described by Lopez-Andreo [19], with minor modifications. Briefly, DNA was isolated from 50 mg of frozen muscle and 100 µL of isolated mitochondria samples using Quick-DNA Prep Kit (Zymo Research, Irvine, CA, USA). DNA concentration was determined using the Nanodrop 2000 (Thermo Scientific, Waltham, MA, USA) to normalize starting DNA concentrations and generate a standard curve with isolated mitochondrial DNA. DNA concentration was normalized and added to TaqMan (Applied Biosystems, Foster City, CA, USA) mix containing species-specific probes and primers for mitochondrial and genomic genes. DNA detection was determined by amplification with 7500 Fast Real-Time PCR System (Applied Biosystems, Foster City, CA, USA). Species-specific reactions were assayed in duplicate with a standard curve on a 96-well plate. Data are displayed as absolute number of mtDNA calculated from the isolated mitochondria DNA standard curve, relative quantity of mtDNA compared to genomic expression (2^−∆CT^), and fold change (2^−∆∆CT^) between muscle types.

### 2.3. SDS-Page and Western Blotting

Whole-cell tissue, isolated mitochondria, and cytosolic fractions were isolated as described by Laker and Drake [20] for SDS-PAGE and Western blotting analyses. Frozen muscle samples were powdered using liquid nitrogen, and a mortar and pestle. Samples were then homogenized using a Polytron PT-MR 2100 homogenizer (Kinematica AG, Malters, Switzerland) with fractionation buffer (20 mM HEPES, 250 mM Sucrose, 0.1 mM EDTA; Sigma-Aldrich, Darmstadt, Germany) at 100 mg/mL concentration containing protease and phosphatase inhibitors (Roche, Basel, Switzerland). Homogenized tissue lysate was then centrifuged at 800× *g* for 10 min at 4 °C. Prior to the centrifugation, an aliquot of this lysate was taken and diluted into a sample buffer (0.5 M Tris-HCl, pH 6.8, 34 mM SDS, 20% Glycerol, 0.1 M DTT, 10% beta-mercaptoethanol; Sigma-Aldrich, Darmstadt, Germany) for the whole cell tissue sample. After centrifugation of the tissue lysate, supernatant was extracted and centrifuged at 9000× *g* for 10 min at 4 °C. The resulting supernatant fractions were re-centrifuged at 17,000× *g* for 10 min at 4 °C to yield the cytosolic fraction, which was also dissolved in the sample buffer. The resulting pellets were washed with fractionation buffer and centrifuged at 11,000× *g* for 10 min at 4 °C to yield the isolated mitochondria pellet, which was then resuspended in a sample buffer. Regardless of tissue or mitochondria, all preparations were heated to 98 °C for 5 min and protein concentration was determined using an RCDC colorimetric protein concentration kit (BioRad, Hercules, CA, USA).

All samples for SDS-PAGE were run on a 10 or 15% poly-acrylamide gel for 30 min at 50 V then 3 h at 100 V. Gels were transferred to nitrocellulose membranes at 35 V overnight at 4 °C. Blots were stained and imaged for total protein concentration with Ponceau S (0.1% Ponceau S in 5%, *v*/*v* acetic acid; Sigma-Aldrich, Darmstadt, Germany) using the ChemiDoc XRS+ imaging system (Bio-Rad, Hercules, CA, USA). Membranes were then blocked with 0.5% nonfat dry milk in 1 X TBST (20 mM Tris base, 140 mM NaCl, and 0.1% Tween; Sigma-Aldrich, Darmstadt, Germany) for 1 h. Primary antibodies against succinate dehydrogenase (SDHA, Abcam 14715, Cambridge, UK), citrate synthase (CS, Santa Cruz Biotechnology 390693, Dallas, TX, USA), electron transport chain complexes cocktail (Abcam 110413, Cambridge, UK), voltage-dependant anion channel (VDAC, Cell Signaling Technology 4461, Danvers, MA, USA), lactate dehydrogenase (LDHA, Novus Biologicals 48336, Centennial, CO, USA), and glyceraldehyde 3-phosphate dehydrogenase (GAPDH, Novus Biologicals 300-221, Centennial, CO, USA) were diluted 1:1000 in blocking buffer and held overnight at 4 °C. Following primary antibody incubation, blots were washed with 1 X TBST three times for 5 min. Secondary antibodies (LI-COR, Lincoln, NE, USA) were added to blots at 1:15,000 dilution for 1 h at 25 °C. Blots were then washed three times with 1 X TBST before being imaged using the Odyssey Scanner (LI-COR, Lincoln, NE, USA). Images were quantified using Image Studio Lite 5.2 (LI-COR, Lincoln, NE, USA). All target proteins were normalized to total protein from Ponceau S stain.

### 2.4. Mitochondrial Isolation

Mitochondria were isolated through differential centrifugation using a protocol adapted from Scheffler et al. [21] with minor modifications. Muscle samples (1:5 wt/vol) were placed into ice-cold homogenization buffer (100 mM sucrose, 180 mM KCl, 50 mM Tris, 10 mM EDTA, 5 mM MgCl2, and 1 mM K-ATP, pH 7.4; Sigma-Aldrich, Darmstadt, Germany, Fisher Scientific, Waltham, MA, USA) and minced finely with dissection scissors. Protease (subtilisin A, P5380, Sigma-Aldrich, Darmstadt, Germany) was added to achieve a final concentration of 0.4 mg/mL and incubated on ice for approximately 10 min. Muscle suspensions were then gently homogenized using a motor-driven Potter-Elvehjem tissue grinder with a teflon pestle and glass mortar (Glas-Col, Terre Haute, IN, USA). Muscle homogenates were then diluted in homogenization buffer to achieve ~20 mL/g, and filtered through two layers of cheesecloth. Filtered muscle suspensions were next centrifuged at 1000× *g* for 10 min at 4 °C. Resulting supernatants were filtered through two layers of cheesecloth and centrifuged at 8000× *g* for 10 min at 4 °C. The resulting mitochondrial pellets were washed and suspended in mannitol sucrose medium (220 mM mannitol, 70 mM sucrose, 10 mM Tris-HCl, and 1 mM EGTA, pH 7.4; Sigma-Aldrich, Darmstadt, Germany). Mitochondrial protein concentration was determined using the bicinchoninc acid protein assay kit (Pierce, Rockford, IL, USA). Finally, isolated mitochondria were diluted to 1 µg/µL with mannitol sucrose medium to determine mitochondrial respiration.

### 2.5. Mitochondrial Respiration

A Seahorse XFe96 (Agilent, Santa Clara, CA, USA) was utilized to quantify mitochondrial respiration following a modified protocol from Boutagy et al. [22]. Substrates used to assess electron transport chain function were: pyruvate/malate (10 mM pyruvic acid and 5 mM malic acid; PyM; Fisher Scientific, Waltham, MA, USA), succinate/rotenone (10 mM succinic acid and 2 µM rotenone; SR; Sigma-Aldrich, Darmstadt, Germany, Fisher Scientific, Waltham, MA, USA), glutamate/malate (10 mM glutamic acid and 5 mM malic acid; GM; Fisher Scientific, Waltham, MA, USA), and palmitoyl-carnitine/malate (40 µM palmitoyl-carnitine and 1 mM malic acid; PCM; Sigma-Aldrich, Darmstadt, Germany; Fisher Scientific, Waltham, MA, USA). Substrates and injections were diluted into mitochondrial assay solution buffer (70 mM Sucrose, 220 mM Mannitol, 5 mM KH_2_PO_4_, 5 mM MgCl_2_, 2 mM HEPES, 1 mM EGTA; Sigma-Aldrich, Darmstadt, Germany; Fisher Scientific, Waltham, MA, USA). Each substrate was prepared individually to contain 0.2% bovine serum albumin (Sigma-Aldrich, Darmstadt, Germany) and to have a pH of 7.4. Diluted fresh mitochondria were loaded onto the Seahorse plate at the following concentrations per well: bovine LL at 1.5 μg and MS at 1 μg for PyM and SR, 2 μg for both muscles for GM, and 1.5 μg for both muscles for PCM; porcine LL at 1.5 μg and MS at 1 μg for PyM, 1 μg for both muscles for SR, 2 μg for GM, and 1.5 μg for PCM; avian PM at 2 μg and QF at 1.5 μg for PyM, 1.5 μg PM and 1.25 μg QF for SR, 2 μg for both muscles for GM and PCM. Concentrations of isolated mitochondria were optimized with a trial plate on each collection day.

Mitochondrial test injections included adenosine diphosphate (ADP; Sigma-Aldrich, Darmstadt, Germany), oligomycin (Tocris Bioscience, Bristol, UK), carbonyl cyanide 4-(trifluoromethoxy) phenylhydrazone (FCCP; Sigma-Aldrich, Darmstadt, Germany), and Antimycin A (Sigma-Aldrich, Darmstadt, Germany). Each stock injection was made individually to achieve 50 mM ADP, 20 µM oligomycin, 40 µM FCCP, and 40 µM Antimycin A. The 50 mM ADP stock injection was prepared with a final pH of 7.4. The final concentrations of each injection per well were: 4 mM ADP, 2 µM Oligomycin, 4 µM FCCP, and 4 µM Antimycin A. ADP was used to stimulate oxidative phosphorylation (OXPHOS) capacity. Oligomycin was utilized to detect the amount of proton leak by inhibiting protons consumed through ATP synthase or complex V. For maximal respiration, FCCP was used to uncouple the membrane potential. To halt all respiratory capacity and show non-mitochondrial respiration, Antimycin A was utilized. Mitochondrial respiration data were normalized to µg of mitochondrial protein loaded per well. Data are displayed to show only mitochondrial oxygen consumption (OCR), which is determined by subtracting non-mitochondrial respiration from all injections.

### 2.6. Statistical Analysis

All data were analyzed using JMP (SAS Institute Inc., Cary, NC, USA) with the animal serving as the experimental unit. Prior to data analysis, normality of residuals was tested using the Shapiro–Wilk test. For data analysis, each statistical model was analyzed separately per species with the main effect of muscle. All data are displayed as least-squares means ± SE. Differences between means of *p* < 0.05 were determined to be significant by using Student’s *t*-test, unless otherwise stated.

## 3. Results

### 3.1. Mitochondrial DNA Copy Number

**Figure 1:**
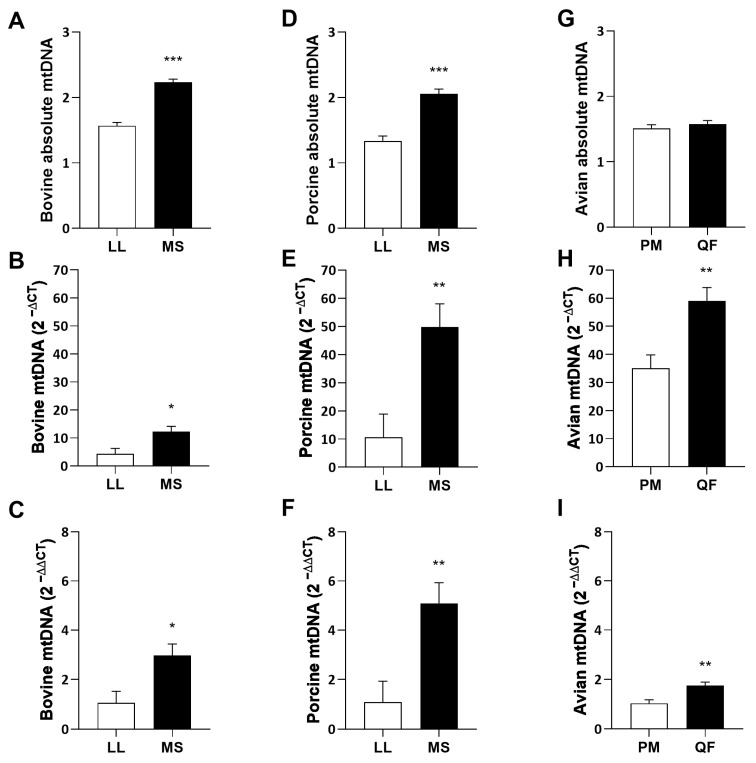
(**A**,**D**,**G**) Absolute mitochondrial DNA (mtDNA) number in glycolytic and oxidative muscles. (**B**,**E**,**H**) Relative mtDNA compared to genomic DNA (2 ^−∆CT^) in glycolytic and oxidative muscles. (**C**,**F**,**I**) Fold change (2 ^−∆∆CT^) of mtDNA in oxidative compared to the glycolytic muscle type. (**A**–**C**) Bovine (*n* = 6) and (**D**–**F**) porcine (*n* = 6) muscle mtDNA content from *longissimus lumborum* (LL) and *masseter* (MS). (**G**–**I**) Avian muscle (*n* = 6) mtDNA content in *pectoralis major* (PM) and *quadriceps femoris* (QF). All values are displayed as least square means followed by standard error bars. Significance is denoted as * *p* < 0.05, ** *p* < 0.01, *** *p* < 0.001.

To measure the abundance of mitochondria in bovine, porcine, and avian skeletal muscles, mitochondrial DNA (mtDNA) abundance was measured. The glycolytic muscles *longissimus lumborum* (LL) and *pectoralis major* (PM) were compared to the oxidative muscles *masseter* (MS) and *quadriceps femoris* (QF) within each species, respectively. Absolute mtDNA number was calculated using isolated mitochondria DNA as the standard, within species (Figure 1A,D,G). To analyze the copy number of mtDNA relative to genomic DNA, data were displayed as the relative mtDNA amounts (2 ^−∆CT^; Figure 1B,E,H) and as a fold change (2 ^−∆∆CT^; Figure 1C,F,I) to compare between glycolytic and oxidative muscles. Bovine (Figure 1A–C) and porcine (Figure 1D–F) MS relative mtDNA content was higher (*p* < 0.05 and *p* < 0.01, respectively) than that of the LL. Avian QF had greater (*p* < 0.01) relative mtDNA (Figure 1H) and fold change (Figure 1I) than PM. However, there were no differences in mitochondrial absolute abundance between avian muscle types (Figure 1G). Regardless of species, oxidative muscles contain greater (*p* < 0.05) fold change of mtDNA than glycolytic muscles (Figure 1C,F,I).

### 3.2. Muscle Protein Abundance

**Figure 2:**
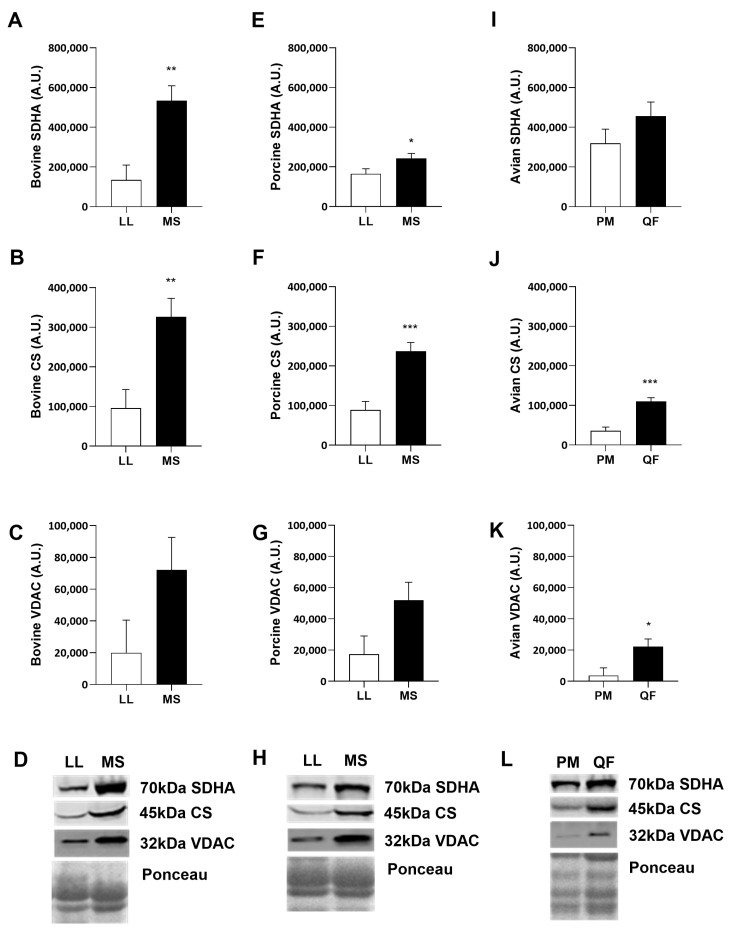
Oxidative protein abundance from whole muscle in bovine (**A**–**D**), porcine (**E**–**H**), and avian (**I**–**L**). Bovine (*n* = 6) and porcine (*n* = 6) muscle protein content from *longissimus lumborum* (LL) and *masseter* (MS). Avian (*n* = 6) muscle protein content in *pectoralis major* (PM) and *quadriceps femoris* (QF). Oxidative protein abundance of (**A**,**E**,**I**) succinate dehydrogenase (SDHA), (**B**,**F**,**J**) citrate synthase (CS), and (**C**,**G**,**K**) voltage-dependent anion channel (VDAC). (**D**,**H**,**L**) Representative Western blot images of SDHA, CS, VDAC, and total protein stain (Ponceau S). All values are displayed as least square means followed by standard error bars. Significance is denoted as * *p* < 0.05, ** *p* < 0.01, *** *p* < 0.001.

To investigate whether the differences in mitochondria are reflected in whole muscle cellular protein content, the following was measured: succinate dehydrogenase (SDHA) as complex II of the electron transport chain, citrate synthase (CS) in the tricarboxylic acid cycle, and voltage-dependent anion channel (VDAC), an outer mitochondrial membrane protein (Figure 2). Western blotting data show that bovine MS muscle contained more SDHA (*p <* 0.01) and CS (*p* < 0.01) compared to its glycolytic counterpart (Figure 2A,B,D). Similarly, porcine MS had increased content of SDHA (*p* < 0.05) and CS, (*p* < 0.001) compared to the LL muscle (Figure 2E,F,H). However, both bovine and porcine muscle had no differences in VDAC abundance (Figure 2C,G) across muscles. Finally, avian QF muscle contained more VDAC (*p* < 0.05) and CS (*p* < 0.001) compared to the PM muscle (Figure 2J–L). Interestingly, there were no detectable differences in SDHA between muscles in avian species (Figure 2I,L).

**Figure 3:**
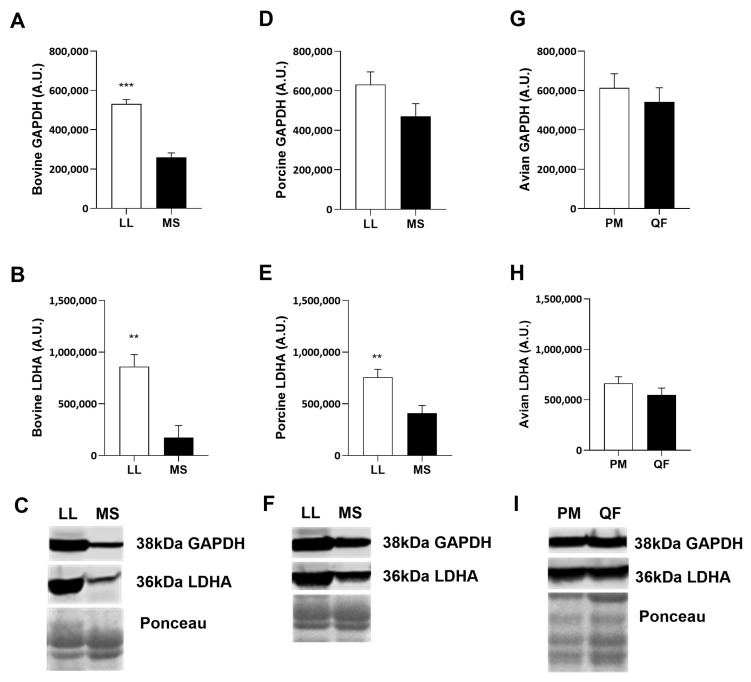
Glycolytic protein abundance from whole muscle in bovine (**A**–**C**), porcine (**D**–**F**), and avian (**G**–**I**). Bovine (*n* = 6) and porcine (*n* = 6) muscle protein content from *longissimus lumborum* (LL) and *masseter* (MS). Avian muscle (*n* = 6) protein content in *pectoralis major* (PM) and *quadriceps femoris* (QF). Glycolytic enzyme protein abundance of (**A**,**D**,**G**) glyceraldehyde 3-phosphate dehydrogenase (GAPDH) and (**B**,**E**,**H**) lactate dehydrogenase (LDHA). (**C**,**F**,**I**) Representative Western blot images of GAPDH, LDHA, and total protein stain (Ponceau S). All values are displayed as least square means followed by standard error bars. Significance is denoted as ** *p* < 0.01 and *** *p* < 0.001.

Additionally, differences were assessed in the glycolytic marker proteins glyceraldehyde-3-phosphate dehydrogenase (GAPDH) and lactate dehydrogenase alpha subunit (LDHA) (Figure 3). Bovine glycolytic LL muscle contained more GAPDH (*p* < 0.001) and LDHA (*p* < 0.01) compared to the MS (Figure 3A–C). Porcine LL muscle had increased (*p* < 0.01) abundance of LDHA compared to porcine MS (Figure 3E,F), but there was no difference in GAPDH abundance (Figure 3D,F). Interestingly, there were no differences in LDHA and GAPDH in avian muscle, regardless of type (Figure 3G–I).

### 3.3. Abundance of Proteins in Mitochondrial Fraction

**Figure 4:**
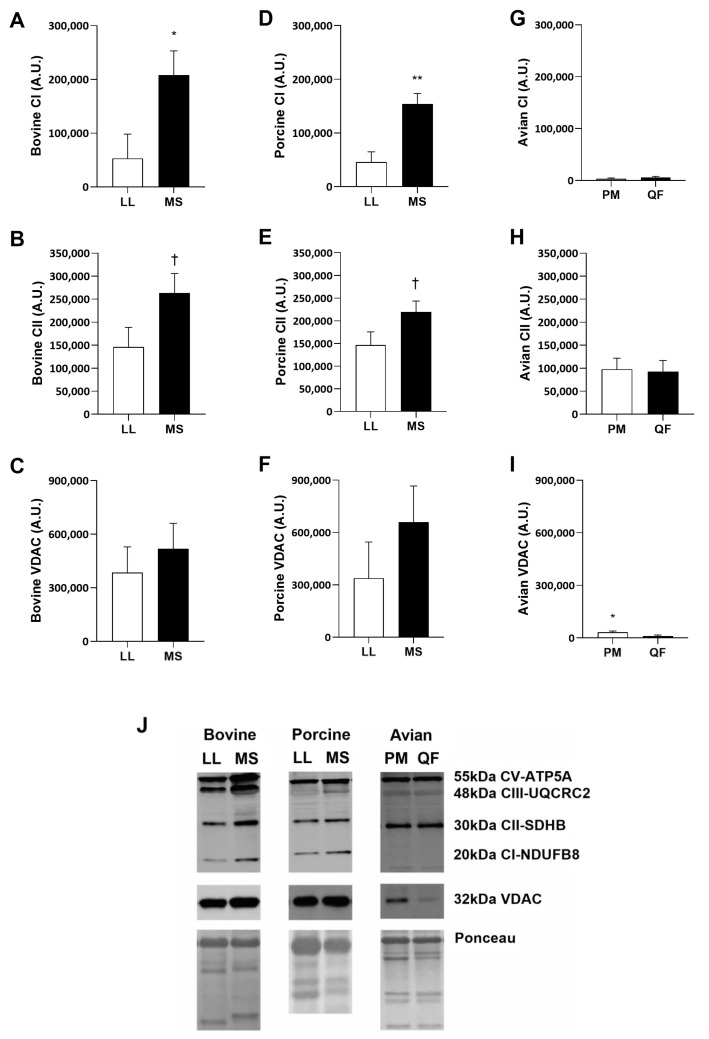
Mitochondrial protein abundance in glycolytic and oxidative muscles from bovine (**A**–**C**), porcine (**D**–**F**), and avian (**G**–**I**) mitochondria enriched fractions. Bovine (*n* = 6) and porcine (*n* = 6) mitochondrial protein content from *longissimus lumborum* (LL) and *masseter* (MS) muscles. Avian (*n* = 6) mitochondrial protein content from *pectoralis major* (PM) and *quadriceps femoris* (QF) muscles. (**A**,**D**,**G**) Mitochondrial proteins abundance of complex I (CI, NDUFB8) and (**B**,**E**,**H**) complex II (CII, SDHB) and (**C**,**F**,**I**) voltage dependent anion channel (VDAC). (**J**) Representative Western blot images of complex I, complex II, complex III, complex V, VDAC, and total protein stain (Ponceau S). All values are displayed as least square means followed by standard error bars. Significance is denoted as † *p =* 0.08, * *p* < 0.05, ** *p* < 0.01.

Enriched mitochondria fractions were used to determine the differences in protein abundance within the mitochondrial electron transport chain (ETC). A cocktail mix of antibodies were used against complex I (CI; NDUFB8), complex II (CII; SDHB), complex III (CIII; UQCRC2), complex IV (CIV; MTCOXI), and complex V (CV; ATP5A). Both bovine and porcine muscle had more CI in MS compared to LL (Figure 4A,D,J, *p* < 0.05 and *p* < 0.01, respectively). Additionally, both bovine and porcine had a greater (*p* = 0.08) CII in MS mitochondria (Figure 4B,E,J). There were no differences in CIII and CV abundance between muscle types in any of the species. Avian mitochondrial enriched portion had no differences in ETC complexes (Figure 4G,H,J). VDAC is typically used as a loading control for enriched mitochondria fractions, and there are no differences between those prepared from bovine or porcine mitochondria fractions (Figure 4C,F). However, there was increased abundance (*p* < 0.05) of VDAC in PM compared to the QF (Figure 4I,J). This is very interesting given VDAC abundance was greater (*p* < 0.05) in the oxidative QF whole muscle sample (Figure 2K). This finding suggests that there are benefits to analyzing proteins on a per mitochondria basis and whole muscle basis.

### 3.4. Mitochondrial Respiration

**Figure 5:**
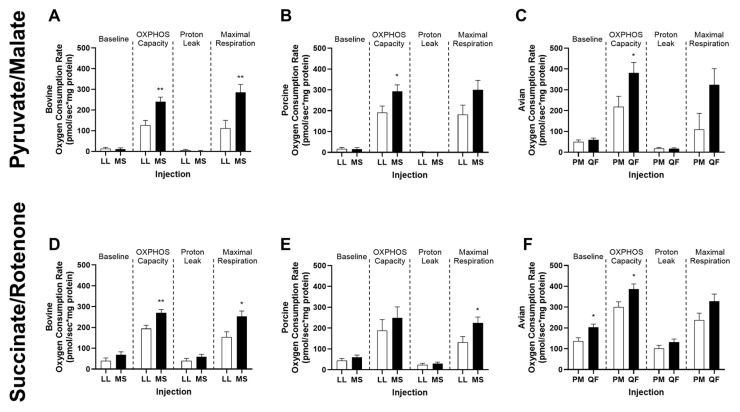
Oxygen consumption rate of mitochondria isolated from (**A**,**D**) bovine (*n* = 6) and (**B**,**E**) porcine (*n* = 6) *longissimus lumborum* (LL) and *masseter* (MS) and (**C**,**F**) avian (*n* = 8) *pectoralis major* (PM) and *quadriceps femoris* (QF) muscles under saturating concentrations of pyruvate/malate (PyM; **A**–**C**) and succinate/rotenone (SR; **D**–**F**) substrates. Baseline represents basal respiration of isolated mitochondria with substrates. OXPHOS capacity is ADP (5 mM) stimulated respiration. Proton leak is determined with 2 µM oligomycin. Maximal respiration is achieved with the uncoupler FCCP (4 µM). All values are displayed as least square means followed by standard error bars. Significance is denoted as * *p* < 0.05 and ** *p* < 0.01.

Mitochondria respiration (oxygen consumption) was measured in vitro to determine the differences in mitochondrial function across glycolytic and oxidative muscles within each species. Bovine mitochondria respiration from MS muscle had greater (*p* < 0.01) oxidative phosphorylation (OXPHOS) capacity compared to those from the LL, regardless of substrate (Figure 5A,D). Additionally, maximal respiration was greater in MS compared to LL mitochondria with pyruvate/malate (Figure 5A, *p* < 0.01) and succinate/rotenone (Figure 5D, *p* < 0.05). Similar to that of bovine, porcine mitochondria also exhibited greater (*p* < 0.05) OXPHOS capacity in the presence of saturating pyruvate/malate (Figure 5B) within the MS muscle. As expected, greater maximal respiration (*p* < 0.05) was also observed in the MS when subjected to succinate/rotenone compared to that of the LL (Figure 5E). Conclusively, these data show both bovine and porcine mitochondria from oxidative tissues have more mitochondrial respiration capacity than mitochondria isolated from glycolytic tissues. Even so, avian mitochondria isolated from PM and QF surprisingly followed similar trends. An increase (*p* < 0.05) in OXPHOS capacity was noted in the QF compared to that of the PM regardless of substrate (Figure 5C,F). Additionally, QF mitochondria also showed an increase (*p* < 0.05) in baseline respiration compared to PM with succinate/rotenone (Figure 5F).

**Figure 6:**
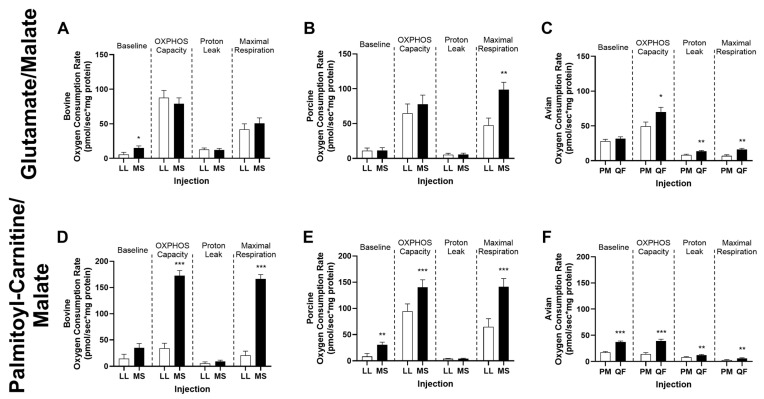
Oxygen consumption rate of mitochondria isolated from (**A**,**D**) bovine (*n* = 6) and (**B**,**E**) porcine (*n* = 6) *longissimus lumborum* (LL) and *masseter* (MS) and (**C**,**F**) avian *pectoralis major* (PM, *n* = 10) and *quadriceps femoris* (QF, *n* = 9) muscles under saturating concentrations of glutamate/malate (GM; **A**–**C**) and palmitoyl-carnitine/malate (PCM; **D**–**F**) substrates. Baseline represents basal respiration of isolated mitochondria with substrates. OXPHOS capacity is ADP (5 mM) stimulated respiration. Proton leak is determined with 2 µM oligomycin. Maximal respiration is achieved with the uncoupler FCCP (4 µM). All values are displayed as least square means followed by standard error bars. Significance is denoted as * *p* < 0.05, ** *p* < 0.01, and *** *p* < 0.001.

Additionally, other substrates were utilized to determine the diverse functionality of mitochondria. Bovine mitochondria respiration from MS muscle had greater (*p* < 0.05) basal respiration than LL muscle when provided with substrates glutamate/malate (GM; Figure 6A). When given palmitoyl-carnitine/malate (PCM), OXPHOS capacity and maximal respiration were greater (*p* < 0.001) in bovine MS mitochondria (Figure 6D). Porcine mitochondria isolated from the MS muscle had greater (*p* < 0.01) maximal respiration under saturating conditions of GM (Figure 6B). Similar to that of bovine, porcine MS mitochondria had greater basal respiration (*p* < 0.01), OXPHOS capacity (*p* < 0.001) and maximal respiration (*p* < 0.001) when given PCM (Figure 6E). In addition, avian oxidative mitochondria had greater OXPHOS capacity (*p* < 0.05), proton leak (*p* < 0.01), and maximal respiration (*p* < 0.01) than glycolytic mitochondria, when provided GM (Figure 6C). When avian mitochondria were provided with PCM (Figure 6F), QF mitochondria had greater oxygen consumption in all injections compared to PM mitochondria (*p* < 0.01).

## 4. Discussion

Interpretation of overall tissue metabolism based on various mitochondrial indicators is difficult. There are several approaches for determining mitochondrial abundance, such as protein abundance and gene expression [12,23,24]. In addition to several means of assessing mitochondrial abundance, there are a wide range of technical difficulties in normalizing such data, which can impact the implications of results. Furthermore, characterizing the contribution of mitochondria to the energy status of a particular cell type requires functional organelle data, which can be evaluated in vitro through oxygen consumption rate using various methods [25]. Isolated mitochondria, intact cells, and muscle fibers have been utilized to measure mitochondrial respiration in vitro [22]. By utilizing mitochondria respiration in vitro, mitochondrial function can be determined to understand its role in muscle. Herein, mtDNA quantity, mitochondrial protein abundance, and mitochondrial respiration were assessed to evaluate the comprehensive function of the mitochondria in two divergent muscle types of three aggressively selected, domestic animals.

### 4.1. Mitochondrial DNA Copy Number

Mitochondria contain their own circular DNA, which can be quantified by amplifying mitochondrial DNA (mtDNA) and normalized to host genomic DNA to determine the mtDNA content within a sample [26,27,28]. However, each mitochondrion may contain multiple copies of mtDNA, whose integrity and abundance are directly linked to mitochondrial function and oxidative phosphorylation (OXPHOS) gene expression capabilities [24,29,30,31]. Fold change (2^−∆∆CT^) was analyzed in mtDNA and showed differences between oxidative and glycolytic muscles. Data within this study showed that the oxidative tissue had more mtDNA than the glycolytic tissue within species, which further validates that mtDNA content differs with muscle type [32]. Interestingly, avian and porcine oxidative tissue had the most relative mtDNA (2^−∆CT^) compared to their glycolytic muscle, while the magnitude of difference was much less between diverse bovine muscles. This could be due to bovine muscles containing greater amounts of oxidative type I and IIa muscle fibers consistently across both glycolytic and oxidative muscle types [33]. In fact, bovine muscle lacks IIb myosin heavy chain expression [34], though classical approaches misidentified a subset of muscle fibers as histologically IIb [35], thus suggesting that bovine skeletal muscle relies more heavily on oxidative metabolism. In terms of fold change (2^−∆∆CT^), bovine and porcine oxidative tissues had 2- and 3-fold greater amounts of mtDNA compared to their glycolytic counterparts, respectively. The least difference was noted in avian muscles, where only a 1-fold increase in mtDNA was noted in oxidative muscle compared to glycolytic muscle. The most intriguing observation was that the absolute mtDNA number did not differ across avian muscles studied. This could be partially due to the fact that glycolytic muscle has more copies of mtDNA per genome and thus differences were lost when calculated from a known standard curve. Regardless, this gap in understanding continues to exist in the literature and remains a technical challenge for determining the total mtDNA content accurately [28,36]. Overall, however, oxidative muscles have more mtDNA than the glycolytic muscle across species, which suggests that mtDNA copy number is a good measure to differentiate muscle types. However, mtDNA content alone cannot justify the overall number of mitochondria within a sample.

### 4.2. Muscle Protein Abundance

Many have characterized the differences in glycolytic and oxidative muscles by analyzing mitochondrial protein abundance and glycolytic enzyme activity [12,13,37,38]. In the present study, bovine and porcine oxidative and glycolytic muscles contain more oxidative or glycolytic protein markers, respectively. This is consistent with previous work showing that porcine glycolytic muscle contains more glycolytic enzyme abundance and activity than its slower-contracting counterpart [38]. Surprisingly, only avian muscle had detectible differences in VDAC, suggesting there may be more demand for metabolite transport in and out of the mitochondria in avian oxidative muscle. Interestingly, avian oxidative muscle has more CS and VDAC, but no differences in other glycolytic markers investigated. The increase in mitochondrial protein correlates with the abundance of type I and IIa fibers in oxidative muscles [39], while the lack of glycolytic protein differences can be related to genetic selection for optimizing growth rate. However, the lack of difference in SDHA between avian muscles suggests that mitochondria may play a critical but alternative role in glycolytic muscles than traditionally thought. Together, these data show that oxidative muscle contains more mitochondrial-specific proteins compared to their glycolytic counterparts, regardless of species, and suggest mitochondrial protein abundance is a good indicator of oxidative metabolism in the skeletal muscle of these species.

### 4.3. Abundance of Proteins in Mitochondrial Fraction

A mitochondrial enriched fraction was used to determine specific differences in mitochondrial electron transport chain (ETC) subunits. This enriched fraction may provide a clearer understanding of protein differences on a per mitochondria protein basis, that may not be detected on a whole muscle cell protein extraction. Unfortunately, complex IV was undetectable within our Western blotting analyses across all species, even with rodent muscle control. No differences in complex III and complex V were detected within each species, suggesting that there was not a protein abundance difference on a per mitochondrial protein basis. Regardless, increased concentrations of complex I in bovine and porcine mitochondria suggest that oxidative muscle mitochondria may have the ability to meet the heightened metabolic energy demands that mitochondria from glycolytic muscle lack. In the avian mitochondrial enriched fraction, there were no differences in ETC complexes, but there was a greater abundance of voltage dependent anion channel (VDAC). VDAC is typically used as a loading control for mitochondrial enriched fractions [20]. Interestingly in the present study, avian whole muscle preparation contained more VDAC in oxidative muscle but within isolated mitochondria, there was more VDAC in the glycolytic sample. This can be explained by a greater distribution of VDAC on the outside of the mitochondria to deliver ATP and anions to and from the mitochondria [40]. Perhaps, avian oxidative muscle contains more VDAC mitochondrial protein on a total protein (per µg) basis, but the isolated mitochondrial data indicated that glycolytic mitochondria contain more VDAC pores to allow ease of signaling metabolites in and out of the mitochondria. It is important to note that not all species contain the same amount of these proteins, possibly due to metabolism differences across species. Because mitochondria protein abundance is only an indirect measure of mitochondrial function, these data should be paired with respiration data to support mitochondria functionality.

### 4.4. Mitochondrial Respiration

To determine mitochondrial function, a mitochondrial functional test was utilized with XFe96 Seahorse, as it determines the efficiency of the electron transport chain of live mitochondria in an environment with saturating amounts of substrates. The use of isolated mitochondria is often overlooked due to the artificial nature of the assay [41]. But this approach allows visualization of the capabilities of the mitochondria under saturating conditions. Data show that analyzing mitochondria function in muscle fibers is more realistic due to the natural biological environment and not as stressed or exposed to calcium release during the isolation process [42,43]. However, it is difficult if not impossible to ensure complete intactness of muscle membranes in such assays. Furthermore, analyzing the mitochondria with Oroboros (O2K) is more suitable for examining ROS production and targeting specific oxygen consumption rates with injections [44]. Due to livestock processing procedures, the isolation of mitochondria is preferred due to the damaging of structural integrity of muscle fibers during the sample collection time. Using the Seahorse oxygen consumption approach is also preferred due to the high throughput capabilities of the apparatus to run samples in a 96-well configuration, allowing more output compared to the O2K [45].

Measuring mitochondria oxygen consumption rates (OCR) by using injected stimulators or inhibitors tests the efficiency of the electron transport chain (ETC). By adding ADP to isolated mitochondria, oxidative phosphorylation (OXPHOS) capacity can be interpreted as the ability of the mitochondria to produce ATP. The use of different substrates targets the efficiency of the whole ETC or determines mitochondrial substrate oxidation capacity [46]. The substrates pyruvate and malate (PyM) were utilized to target the whole ETC function from the end product of glycolysis, while succinate and rotenone (SR) were utilized to target ETC complex II through complex V efficiency [22]. These two substrates (SR) were utilized as a basis for analyzing mitochondria respiration, excluding complex I, to better understand the contribution of complex II ETC functionality and abundance. Because agriculturally relevant species are typically fed high-carbohydrate diets, PyM and SR substrates were utilized to determine mitochondrial function based on the end product of glycolysis. However, there are alternative substrates that can be utilized to determine substrate oxidation between carbohydrate and fat sources, which give a more complete understanding of the contribution of mitochondria to metabolism [46]. Glutamate and malate (GM) were utilized to target the ability of mitochondria to generate energy through the use of the glutamate-aspartate carriers for animo acids, while also determining the efficiency of ETC [22,44]. Mitochondria are also the site for beta oxidation; thus, utilization of fatty acids as a substrate is useful in this regard. The substrate palmitoyl-carnitine/malate (PCM) was utilized to assess the ability of long chain fatty acids to be oxidized in the mitochondria [22,47]. Because mitochondria contribute to metabolism by using a number of precursors, many substrates were utilized to give an encompassing overview of mitochondrial function.

Within the constructs of the current study, mitochondria with greater functional capacity (OCR) generally came from more oxidative tissue, regardless of species. Respiration of isolated bovine mitochondria was greater in oxidative muscle compared to glycolytic tissue, yet there was only a significant increase in SDHA, CS, CI, and CII abundance and not other mitochondrial proteins. This could be due to the fact that bovine muscle is more oxidative in nature than other species, regardless of muscle type [33]. Despite porcine oxidative mitochondria having more SDHA in muscle, and SDHB in isolated mitochondria, the respiration efficiency in saturating amounts of SR is not different for their OXPHOS capacity. This directly shows that complex II protein abundance is not linked to the function of the mitochondria. Similarly, a study in mouse skeletal muscle revealed no correlation between SDHA abundance and mitochondria respiration; however, a correlation between SDHB abundance and mitochondria respiration was noted [48]. However, mitochondria from porcine oxidative muscle had increased OXPHOS capacity when provided saturating amounts of PyM. These data suggest complex I has an additive contribution to the overall OXPHOS capacity when combined with complex II. In addition, the ETC can be assessed with other substrates to determine the flexibility of the mitochondria to process differing substrates. Bovine mitochondria from red or white muscle have no differences in respiration when provided GM, which suggests that the ability of mitochondria to utilize GM is not different between muscle types. However, when provided with PCM, a drastic difference was noted in the ability of oxidative mitochondria to utilize fatty acid substrate compared to those mitochondria isolated from glycolytic muscle. Together, these data demonstrate the importance of using different substrates to determine the wholistic contribution of the mitochondria to muscle metabolism across different muscle types.

Interestingly, there was no difference in the maximal respiration between muscles when using purified avian mitochondria provided with PyM and SR substrates. This suggests that avian mitochondria are not different in their ability to couple ETC proton gradient with ATP production. Essentially, avian mitochondria have an ability to produce more ATP in the oxidative tissue but, overall, their ability to couple the proton gradient to ATP is not different, suggesting that mitochondria functional efficiency is not necessary for glycolytic metabolism. However, when avian mitochondria are provided GM and PCM, the mitochondria from oxidative muscle have a greater OCR than those from glycolytic muscle. This underscores the importance of using multiple substrates to study the diverse functionality of the mitochondria. Taken in totality, our data suggest avian mitochondria in glycolytic muscles contribute differently to the overall metabolism of the tissue compared to those from more oxidative muscles. Indeed, mitochondria function depends on the muscle type and species, and these differences and the reasons why they exist warrant further investigation.

## 5. Conclusions

Overall, mitochondria abundance and function vary with muscle type within species. Oxidative metabolism in muscle relies on the amount of mtDNA present, the abundance of mitochondrial proteins and the capacity of the mitochondria to function in response to a variety of specific substrates. However, differences in the abundances of mtDNA and mitochondrial proteins exist across species, either on a whole muscle or mitochondria-enriched fraction basis. Our findings confirm the importance of evaluating mitochondrial content and function when studying the role of this organelle’s contribution to overall muscle metabolism. Most notably, within species and across skeletal muscle types, mtDNA abundance and mitochondria proteins are not necessarily related to overall mitochondria function.

## Data Availability

The data presented in this study are available on request from the corresponding author due to privacy.

## References

[1] Capper J.L. (2011). The environmental impact of beef production in the United States: 1977 compared with 2007. J. Anim. Sci..

[2] Terry S.A., Basarab J.A., Guan L.L., McAllister T.A. (2021). Strategies to improve the efficiency of beef cattle production. Can. J. Anim. Sci..

[3] Wu F., Vierck K.R., DeRouchey J.M., O’Quinn T.G., Tokach M.D., Goodband R.D., Dritz S.S., Woodworth J.C. (2017). A review of heavy weight market pigs: Status of knowledge and future needs assessment. Transl. Anim. Sci..

[4] Zuidhof M.J., Schneider B.L., Carney V.L., Korver D.R., Robinson F.E. (2014). Growth, efficiency, and yield of commercial broilers from 1957, 1978, and 2005. Poult. Sci..

[5] Drouillard J.S. (2018). Current situation and future trends for beef production in the United States of America—A review. Asian-Australas. J. Anim. Sci..

[6] Wicks J., Beline M., Gomez J.F.M., Luzardo S., Silva S.L., Gerrard D. (2019). Muscle Energy Metabolism, Growth, and Meat Quality in Beef Cattle. Agriculture.

[7] Schiaffino S., Reggiani C. (1996). Molecular diversity of myofibrillar proteins: Gene regulation and functional significance. Physiol. Rev..

[8] Schiaffino S., Reggiani C. (2011). Fiber Types in Mammalian Skeletal Muscles. Physiol. Rev..

[9] Arany Z., Lebrasseur N., Morris C., Smith E., Yang W., Ma Y., Chin S., Spiegelman B.M. (2007). The Transcriptional Coactivator PGC-1β Drives the Formation of Oxidative Type IIX Fibers in Skeletal Muscle. Cell Metab..

[10] Bottje W.G., Carstens G.E. (2012). Variation in metabolism: Biological efficiency of energy production and utilization that affects feed efficiency. Feed Efficiency in the Beef Industry.

[11] Mishra P., Varuzhanyan G., Pham A.H., Chan D.C. (2015). Mitochondrial Dynamics is a Distinguishing Feature of Skeletal Muscle Fiber Types and Regulates Organellar Compartmentalization. Cell Metab..

[12] Glancy B., Balaban R.S. (2011). Protein composition and function of red and white skeletal muscle mitochondria. Am. J. Physiol.-Cell Physiol..

[13] Apaoblaza A., Gerrard S.D., Matarneh S.K., Wicks J.C., Kirkpatrick L., England E.M., Scheffler T.L., Duckett S.K., Shi H., Silva S.L. (2020). Muscle from grass- and grain-fed cattle differs energetically. Meat Sci..

[14] Morales Gómez J.F., Antonelo D.S., Beline M., Pavan B., Bambil D.B., Fantinato-Neto P., Saran-Netto A., Leme P.R., Goulart R.S., Gerrard D.E. (2022). Feeding strategies impact animal growth and beef color and tenderness. Meat Sci..

[15] Antonelo D.S., Gómez J.F.M., Silva S.L., Beline M., Zhang X., Wang Y., Pavan B., Koulicoff L.A., Rosa A.F., Goulart R.S. (2022). Proteome basis for the biological variations in color and tenderness of longissimus thoracis muscle from beef cattle differing in growth rate and feeding regime. Food Res. Int..

[16] Rehfeldt C., Henning M., Fiedler I. (2008). Consequences of pig domestication for skeletal muscle growth and cellularity. Livest. Sci..

[17] Reverter A., Okimoto R., Sapp R., Bottje W.G., Hawken R., Hudson N.J. (2016). Chicken muscle mitochondrial content appears co-ordinately regulated and is associated with performance phenotypes. Biol. Open.

[18] Zeng C., Shi H., Kirkpatrick L.T., Ricome A., Park S., Scheffler J.M., Hannon K.M., Grant A.L., Gerrard D.E. (2022). Driving an Oxidative Phenotype Protects Myh4 Null Mice From Myofiber Loss During Postnatal Growth. Front. Physiol..

[19] López-Andreo M., Lugo L., Garrido-Pertierra A., Prieto M.I., Puyet A. (2005). Identification and quantitation of species in complex DNA mixtures by real-time polymerase chain reaction. Anal. Biochem..

[20] Laker R.C., Drake J.C., Wilson R.J., Lira V.A., Lewellen B.M., Ryall K.A., Fisher C.C., Zhang M., Saucerman J.J., Goodyear L.J. (2017). Ampk phosphorylation of Ulk1 is required for targeting of mitochondria to lysosomes in exercise-induced mitophagy. Nat. Commun..

[21] Scheffler T.L., Matarneh S.K., England E.M., Gerrard D.E. (2015). Mitochondria influence postmortem metabolism and pH in an in vitro model. Meat Sci..

[22] Boutagy N.E., Pyne E., Rogers G.W., Ali M., Hulver M.W., Frisard M.I. (2015). Isolation of Mitochondria from Minimal Quantities of Mouse Skeletal Muscle for High Throughput Microplate Respiratory Measurements. J. Vis. Exp..

[23] Herbst A., Widjaja K., Nguy B., Lushaj E.B., Moore T.M., Hevener A.L., McKenzie D., Aiken J.M., Wanagat J. (2017). Digital PCR Quantitation of Muscle Mitochondrial DNA: Age, Fiber Type, and Mutation-Induced Changes. J. Gerontol. Ser. A Biol. Sci. Med. Sci..

[24] Cole L.W. (2016). The Evolution of Per-cell Organelle Number. Front. Cell Dev. Biol..

[25] Hoeks J., Hesselink M., Schrauwen P., Mooren F.C. (2012). Mitochondrial Respiration. Encyclopedia of Exercise Medicine in Health and Disease.

[26] Groot G.S.P., Kroon A.M. (1979). Mitochondrial DNA from various organisms does not contain internally methylated cytosine in -CCGG- sequences. Biochim. Biophys. Acta (BBA)—Nucleic Acids Protein Synth..

[27] Malik A.N., Shahni R., Iqbal M.M. (2009). Increased peripheral blood mitochondrial DNA in type 2 diabetic patients with nephropathy. Diabetes Res. Clin. Pract..

[28] Malik A.N., Czajka A. (2013). Is mitochondrial DNA content a potential biomarker of mitochondrial dysfunction?. Mitochondrion.

[29] Ajaz S., Czajka A., Malik A., Weissig V., Edeas M. (2015). Accurate Measurement of Circulating Mitochondrial DNA Content from Human Blood Samples Using Real-Time Quantitative PCR. Mitochondrial Medicine: Volume I, Probing Mitochondrial Function.

[30] Hock M.B., Kralli A. (2009). Transcriptional Control of Mitochondrial Biogenesis and Function. Annu. Rev. Physiol..

[31] Williams R.S. (1986). Mitochondrial gene expression in mammalian striated muscle. Evidence that variation in gene dosage is the major regulatory event. J. Biol. Chem..

[32] Veltri K.L., Espiritu M., Singh G. (1990). Distinct genomic copy number in mitochondria of different mammalian organs. J. Cell. Physiol..

[33] Song S., Ahn C.H., Kim G.D. (2020). Muscle Fiber Typing in Bovine and Porcine Skeletal Muscles Using Immunofluorescence with Monoclonal Antibodies Specific to Myosin Heavy Chain Isoforms. Food Sci. Anim. Resour..

[34] Tanabe R.I., Susumu M., Koichi C. (1998). Sequencing of the 2a, 2x, and slow isoforms of the bovine myosin heavy chain and the different expression among muscles. Mamm. Genome.

[35] Wegner J., Albrecht E., Fiedler I., Teuscher F., Papstein H.-J., Ender K. (2000). Growth- and breed-related changes of muscle fiber characteristics in cattle1. J. Anim. Sci..

[36] Hammond E.L., Sayer D., Nolan D., Walker U.A., Ronde A., Montaner J.S., Cote H.C., Gahan M.E., Cherry C.L., Wesselingh S.L. (2003). Assessment of precision and concordance of quantitative mitochondrial DNA assays: A collaborative international quality assurance study. J. Clin. Virol..

[37] Klont R.E., Brocks L., Eikelenboom G. (1998). Muscle fibre type and meat quality. Meat Sci..

[38] England E.M., Matarneh S.K., Oliver E.M., Apaoblaza A., Scheffler T.L., Shi H., Gerrard D.E. (2016). Excess glycogen does not resolve high ultimate pH of oxidative muscle. Meat Sci..

[39] Ono Y., Iwamoto H., Takahara H. (1993). The Relationship Between Muscle Growth and the Growth of Different Fiber Types in the Chicken. Poult. Sci..

[40] Noskov S.Y., Rostovtseva T.K., Chamberlin A.C., Teijido O., Jiang W., Bezrukov S.M. (2016). Current state of theoretical and experimental studies of the voltage-dependent anion channel (VDAC). Biochim. Biophys. Acta (BBA) Biomembr..

[41] Schuh R.A., Jackson K.C., Khairallah R.J., Ward C.W., Spangenburg E.E. (2011). Measuring mitochondrial respiration in intact single muscle fibers. Am. J. Physiol. Regul. Integr. Comp. Physiol..

[42] Picard M., Taivassalo T., Gouspillou G., Hepple R.T. (2011). Mitochondria: Isolation, structure and function. J. Physiol..

[43] Lanza I.R., Nair K.S. (2009). Functional assessment of isolated mitochondria in vitro. Methods Enzymol..

[44] Gnaiger E. (2009). Capacity of oxidative phosphorylation in human skeletal muscle: New perspectives of mitochondrial physiology. Int. J. Biochem. Cell Biol..

[45] Horan M.P., Pichaud N., Ballard J.W.O. (2012). Review: Quantifying Mitochondrial Dysfunction in Complex Diseases of Aging. J. Gerontol. Ser. A.

[46] Leverve X.M., Fontaine E. (2001). Role of Substrates in the Regulation of Mitochondrial Function In Situ. Int. Union Biochem. Mol. Biol..

[47] Kerner J., Hoppel C. (2000). Fatty acid import into mitochondria. Biochim. Biophys. Acta (BBA) Mol. Cell Biol. Lipids.

[48] Maekawa S., Takada S., Furihata T., Fukushima A., Yokota T., Kinugawa S. (2020). Mitochondrial respiration of complex II is not lower than that of complex I in mouse skeletal muscle. Biochem. Biophys. Rep..

